# Relationship between serum ferritin and the risk of all-cause death in patients with coronary artery disease

**DOI:** 10.1016/j.clinsp.2025.100772

**Published:** 2025-09-02

**Authors:** Zhi-Hui Jiang, Ming-Ming Lyu, Yu-Juan Feng, Ting-Ting Wu, Ying Pan, Chang-Jiang Deng, Yi Yang, Xiao-Xia Guo, Ying-Ying Zheng, Xiang Xie

**Affiliations:** a Department of Cardiology, First Affiliated Hospital of Xinjiang Medical University, Urumqi, China; b Key Laboratory of High Incidence Disease Research in Xingjiang, Xinjiang Medical University, Ministry of Education, Urumqi, China; c Key Laboratory of Hypertension Research of Xinjiang Medical University, Urumqi, China

**Keywords:** Ferritin, Mortality, Coronary artery disease, Predictor

## Abstract

**Objective:**

Ferritin is independently associated with oxidized Low-Density Lipoprotein (LDL). While high LDL levels are known independent risk factors for cardiovascular diseases, few studies have investigated the relationship between serum ferritin levels and long-term outcomes in CAD patients.

**Methods:**

A prospective cohort study of CAD participants was performed in Xinjiang Medical University Affiliated First Hospital from December 2016 to October 2021. 1089 CAD patients were divided into two groups according to baseline serum ferritin concentration (ferritin < 160, *n* = 715 and ferritin ≥ 160, *n* = 374). The authors investigated long-term mortality as the primary endpoint, including All-Cause Mortality (ACM) and Cardiac Mortality (CM). The longest follow-up duration was 60 months.

**Results:**

In this study, a total of 17 ACMs, 12 CMs, 34 MACEs, and 41 MACCEs were recorded. There were significant differences between the two groups in the incidence of ACM (*p* = 0.002) and CM (*p* = 0.019). After controlling for covariates, multivariate Cox regression analysis showed that ferritin was an independent predictor ACM (Hazard Ratio [HR = 2.674], 95 % Confidence Interval [95 % CI 1.197‒5.971], *p* = 0.016) in CAD patients.

**Conclusions:**

High ferritin levels are strongly associated with a higher risk of ACM in CAD patients.

## Highlights

• High ferritin levels are linked to increased all-cause mortality risk in CAD patients.
• Elevated ferritin is associated with a 167.4 % higher risk of all-cause mortality.
• Long follow-up ensures reliable findings, supporting ferritin as a risk stratification tool.

## Introduction

Coronary Artery Disease (CAD) is a major global public health burden, with rising annual morbidity and mortality pose a serious threat to human health.^1^, ^2^, ^3^, ^4^ Iron is essential for cellular functions in living organisms and catalyzes the formation of potentially toxic free radicals. Excess iron is stored in cells by ferritin, as a nontoxic and readily available form.^5^, ^6^, ^7^ Ferritin and iron exhibit pro-oxidant properties, with ferritin serving as an independent positive determinant of oxidized LDL levels. A study conducted in Pakistan demonstrated that serum ferritin levels were significantly higher in patients with CAD compared to those without CAD.^8^ Serum ferritin was found to be associated with increasing cardiovascular risk in the Iranian male population, and it is a strong and independent risk factor for atherosclerotic events.^9^^,^^10^ However, the predictive value of serum ferritin in cardiovascular disease is still controversial. Some experts argue that ferritin does not predict the risk for CAD. A study indicated that serum concentrations of ferritin do not predict the risk for CAD. Among subjects with preexisting CAD, those with more severe disease exhibited decreased levels of ferritin. Conversely, CAD patients presenting with acute coronary syndromes demonstrated increased levels of serum ferritin.^11^ The latest Mendelian Randomization (MR) analyses showed that higher levels of iron biomarkers were protective for CAD, had adverse effects on type 2 diabetes, but had no effects on Ischemic Stroke (IS) or Heart Failure (HF).^12^ Therefore, additional studies may be required to explore the predictive value of ferritin on cardiovascular events.

To the best of our knowledge, the relationship between ferritin levels and outcomes in CAD patients has not been previously investigated. In this study, the authors enrolled 1089 CAD patients and conducted a long-term follow-up to examine the correlation between ferritin levels and clinical outcomes.

## Method

### Study design and patients

All patients were from the PRACTICE study, a large single-center prospective cohort study performed in the First Affiliated Hospital of Xinjiang Medical University from December 2016 to October 2021. Details of the study design have been registered on http://www.chictr.org.cn (identifier: NCT05174143). Given that ferritin assays are not routinely performed, the authors enrolled 1089 CAD patients with available ferritin data from the PRACTICE study. The inclusion criteria were CAD patients who were undergoing coronary angiography, with coronary stenosis ≥ 70 %. The authors excluded patients with serious heart failure, rheumatic heart disease, valvular heart disease, congenital heart disease, pulmonary heart disease, serious dysfunction of the liver or kidney, or unavailable serum ferritin data. The study protocol was approved by the ethics committee of the First Affiliated Hospital of Xinjiang Medical University and followed the STROBE Statement. Patients provided informed consent to participate.

**Fig. 1:**
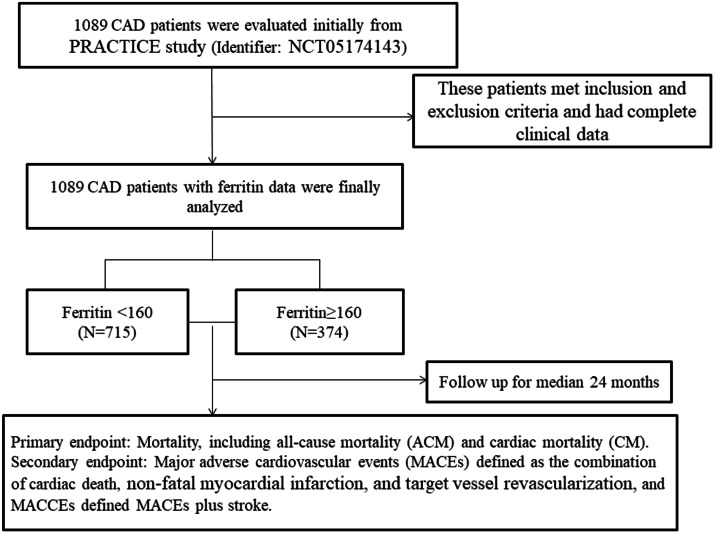
The flow chart of the inclusion and exclusion criteria.

To investigate the relationship between serum ferritin and outcomes in patients with CAD, a total of 1089 patients were initially evaluated. These patients met the inclusion and exclusion criteria and had complete clinical data. All patients were divided into two groups according to dichotomy of serum ferritin, which was based on the ROC cut-off: the low-level group (ferritin < 160, *n* = 715); the high-level group (ferritin ≥ 160, *n* = 374). Fig. 1 shows the flow chart of the inclusion and exclusion criteria.

### Data collection

The authors collected and recorded demographic data of patients, including age, sex, smoking, alcohol consumption, familial history of CAD, previous diagnosis of diabetes, history of hypertension, and history of medication. Fasting blood samples were carried out before coronary angiography after at least 12 h of fasting. Creatinine (Cr), Uric Acid (UA), Triglyceride (TG), Total Cholesterol (TC), Low-Density Lipoprotein-C (LDL-C), and High-Density Lipoprotein-C (HDL-C) were measured in the Medical Laboratory Centre of the First Affiliated Hospital of Xinjiang Medical University. Medications were also collected by medical records review and self-report.

### Endpoints

The primary endpoint of this study was to assess mortality, consisting of All-Cause Mortality (ACM), and Cardiac Mortality (CM). The secondary endpoints were Major Adverse Cardiovascular Events (MACEs), defined as a combination of cardiac death, non-fatal myocardial infarction, and target vessel revascularization, and Major Adverse Cardiovascular and Cerebrovascular Events (MACCEs), defined as MACE plus stroke. As for target vessel revascularization, it is defined as any repetitive revascularization of a treated vessel with a stenosis diameter of at least 50 % in the presence of ischemic signs or symptoms, or at least 70 % in the absence of ischemic signs or symptoms.^13^

### Follow-up

In this study, all CAD patients have received regular follow-up after discharge by office visits, telephone interviews, or questionnaire surveys at the end of 1-month, 3-months, 6-months, 1-year, 3-years, and 5-years. Patients were followed up for at least 6-months, and the longest follow-up time was 5-years. Trained clinical physicians would assess the compliance of the drugs and adverse events carefully.

### Statistical analyses

All analyses were performed using SPSS 26.0 (SPSS Inc., Chicago, IL, USA). Serum ferritin was analyzed as a continuous variable and categorized into two groups based on the cut-off value of 160. The cut-off value (160) was determined according to the ROC curve analysis for the baseline data of the study population, maximizing both sensitivity and specificity. Continuous variables were presented as the mean ± standard deviation, and categorical variables were presented as the frequencies and percentages. Student’s *t*-tests were used to compare parametric continuous variables, and Mann-Whitney *U* tests were used to compare non-parametric continuous variables. Chi-Squared tests were used to compare categorical variables. Kaplan-Meier analysis was used for cumulative incidence rates of long-term outcomes, and the log-rank test was used for comparisons between groups. After adjusting for the influence of age, alcohol drinking, TG, HDL-C, multivariate Cox regression analysis was used to compute Hazard Ratios (HRs) and 95 % CIs for adverse outcomes. The p-values were 2-sided, and *p* < 0.05 was considered statistically significant.

## Results

### Baseline characteristics of patients

In this study, the authors divided the enrolled patients into two groups according to the serum ferritin value. Baseline characteristics of both groups are shown in Table 1. There were 715 patients in the low ferritin group and 374 patients in the high ferritin group. Participants with higher ferritin values were predominantly male, had a higher incidence of alcohol drinking, and exhibited higher levels of TG. Higher level of HDL-C was found in the low ferritin group (all *p* < 0.05). However, there were no significant differences between the two groups in terms of age, smoking incidence, levels of Cr, UA, TC, LDL-C, Ejection Fraction (EF), usage of Calcium Channel Blockers (CCB), β-blockers, Renin-Angiotensin System Inhibitors (RASI), aspirin, substitution of clopidogrel with ticagrelor, and statins (all *p* > 0.05).

**Table 1:** Characteristics of participants of the two groups.

Variables	Ferritin < 160 (n = 715)	Ferritin ≥ 160 (n = 374)	X^2^or *t*	p-value
Age, years	61.89 ± 11.91	61.06 ± 11.93	1.098	0.272
Sex, Male, n (%)	465 (65.0)	299 (79.9)	26.078	**<0.001**
Smoking, n (%)	279 (39.0)	165 (44.1)	2.641	0.104
Alcohol drinking, n (%)	144 (20.1)	103 (27.5)	7.668	**0.006**
Cr, umoL/L	86.42 ± 115.11	92.52 ± 75.69	−0.880	0.379
UA, mmoL/L	555.04 ± 831.30	615.07 ± 910.32	−1.094	0.274
TG, mmoL/L	1.73 ± 1.28	1.99 ± 1.67	−2.659	**0.008**
TC, mmoL/L	3.91 ± 1.13	3.89 ± 1.09	0.336	0.737
LDL-C, mmoL/L	2.45 ± 0.89	2.49 ± 0.84	−0.749	0.454
HDL-C, mmoL/L	1.10 ± 0.33	1.02 ± 0.29	3.820	**<0.001**
EF, (%)	59.74 ± 8.79	59.48 ± 8.47	0.466	0.642
CCB, n (%)	182 (26.5)	79 (21.8)	2.723	0.099
β-blockers, n (%)	418 (60.7)	199 (55.1)	3.004	0.083
RASI, n (%)	340 (47.6)	193 (51.6)	1.613	0.204
Aspirin, n (%)	677 (94.7)	350 (350)	0.556	0.456
Ticagrelor, n (%)	371 (51.9)	194 (51.9)	<0.001	0.996
Statins, n (%)	658 (92.0)	346 (92.5)	0.080	0.777

BUN, Blood Urea Nitrogen; UA, Uric Acid; Cr, Creatinine; GLU, Glucose; TG, Triglyceride; TC, Total Cholesterol; LDL-C, Low Density Lipoprotein Cholesterol; HDL-C, High Density Lipoprotein Cholesterol; EF, Ejection Fraction; CCB, Calcium Channel Blockers; RASI, Renin-Angiotensin System Inhibitors.

### Clinical outcomes

As shown in Table 2, for the primary endpoints, the incidence of ACM in the low ferritin group was 17 (2.4 %), compared to 23 (6.1 %) in the high ferritin group, with a significant difference (*p* = 0.002). Similarly, the incidence of CM between the two groups also differed significantly (1.7 % vs. 4.0 %, *p* = 0.019). For the secondary endpoints, the authors found that there were no significant differences between the two groups in the incidence of MACCEs (5.7 % vs. 8.3 %, *p* = 0.107), MACEs (4.8 % vs. 7.5 %, *p* = 0.065).

**Table 2:** Outcomes comparison between groups.

Outcomes	Ferritin < 160 (n = 715)	Ferritin ≥ 160 (n = 374)	X^2^	p-value
ACM, n (%)	17 (2.4)	23 (6.1)	9.875	**0.002**
CM, n (%)	12 (1.7)	15 (4.0)	5.525	**0.019**
MACCEs, n (%)	41 (5.7)	31 (8.3)	2.595	0.107
MACEs, n (%)	34 (4.8)	28 (7.5)	3.412	0.065

ACM, All-Cause Mortality; CM, Cardiac Mortality; MACCEs, Major Adverse Cardiovascular and Cerebrovascular Events; MACEs, Major Adverse Cardiovascular Events.

**Fig. 2:**
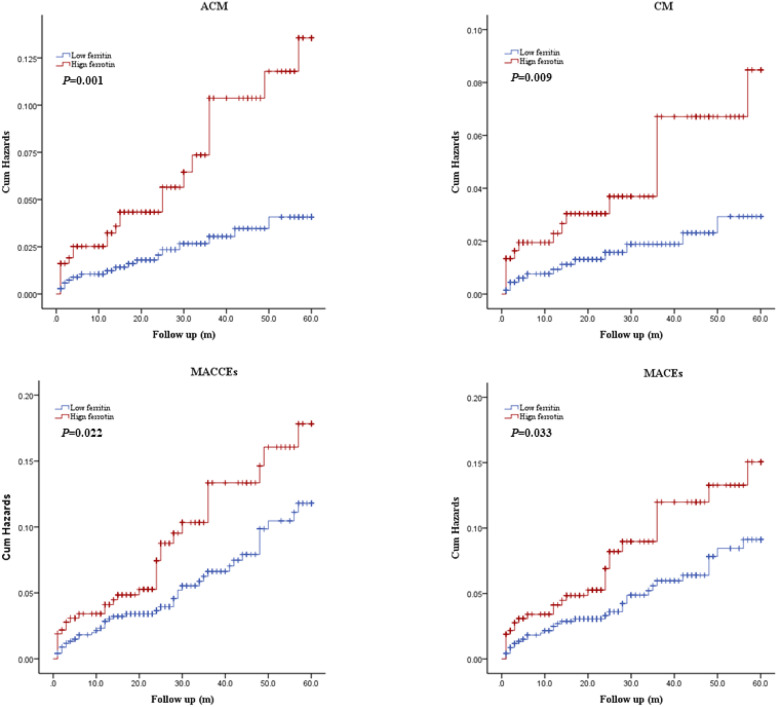
Cumulative Kaplan-Meier estimates of the time to the first adjudicated occurrence of primary endpoints and secondary endpoints: low ferritin for ferritin < 160, and high ferritin for ferritin ≥ 160. ACM, All-Cause Mortality; CM, Cardiac Mortality; MACCEs, Major Adverse Cardiovascular and Cerebrovascular Events; MACEs, Major Adverse Cardiovascular Events.

Kaplan-Meier curves for serum ferritin divided by adverse outcomes are shown in Fig. 2, indicating that patients with high ferritin levels exhibited a significantly increased cumulative risk of adverse clinical events (all *p* < 0.05).

Univariate models were created for each of the predictor variables, and variables that were significant (*p* < 0.05) in univariate Cox models were entered into multivariate Cox regression analysis. As shown in Table 3, after adjustment for other confounders, such as age, alcohol drinking, TG, HDL-C, serum ferritin remained associated with ACM. That is, compared with the risks for patients in the high ferritin group, the risk for all cause death was increased by 167.4 % (HR = 2.674, 95 % CI 1.197‒5.971, *p* = 0.016), however, there were no significant difference between the two groups in the incidence of CM, MACCEs, or MACEs (all *p* > 0.05).

**Table 3:** Multivariable Cox regression analysis for outcomes.

Variables	B	SE	Wald	p-values	HR (95 % CI)
**ACM**					
Age	−0.490	0.573	0.732	0.392	0.612 (0.199‒1.884)
Alcohol drinking	−0.003	0.457	0	0.995	0.997 (0.407‒2.443)
TG	−0.691	0.325	4.531	**0.033**	0.501 (0.265‒0.947)
HDL-C	−0.570	0.71	0.643	0.423	0.566 (0.141‒2.277)
Ferritin	0.983	0.41	5.756	**0.016**	2.674 (1.197‒5.971)
**CM**					
Age	−0.042	0.623	0.005	0.946	0.959 (0.283‒3.251)
Alcohol drinking	−0.021	0.600	0.001	0.973	0.980 (0.302‒3.177)
TG	−0.907	0.459	3.907	**0.048**	0.404 (0.164‒0.992)
HDL-C	−0.242	0.843	0.082	0.774	0.785 (0.151‒4.095)
Ferritin	0.823	0.513	2.579	0.108	2.278 (0.834‒6.220)
**MACCEs**					
Age	0.526	0.303	3.013	0.083	1.692 (0.934‒3.065)
Alcohol drinking	−0.044	0.342	0.017	0.898	0.957 (0.489‒1.871)
TG	0.066	0.08	0.678	0.410	1.068 (0.913‒1.249)
HDL-C	0.073	0.413	0.031	0.859	1.076 (0.479‒2.417)
Ferritin	0.474	0.278	2.906	0.088	1.606 (0.932‒2.768)
**MACEs**					
Age	0.335	0.339	0.982	0.322	1.399 (0.720‒2.716)
Alcohol drinking	0.092	0.351	0.069	0.792	1.097 (0.552‒2.181)
TG	0.071	0.083	0.724	0.395	1.074 (0.912‒1.264)
HDL-C	−0.095	0.468	0.041	0.839	0.909 (0.363‒2.276)
Ferritin	0.438	0.299	2.141	0.143	1.550 (0.862‒2.787)

TG, Triglyceride; HDL-C, High Density Lipoprotein Cholesterol; ACM, All-Cause Mortality; CM, Cardiac Mortality; MACCEs, Major Adverse Cardiovascular and Cerebrovascular Events; MACEs, Major Adverse Cardiovascular Events.

## Discussion

In this study, the authors found that increased serum ferritin was independently associated with all-cause death in patients with CAD. To the best of our knowledge, this is the first study to investigate the relationship between serum ferritin levels and long-term outcomes in CAD patients.

Iron homeostasis is strictly regulated at both the systemic and cellular levels by complex mechanisms due to its indispensability and toxicity. Among the various iron-regulatory proteins, ferritin is the earliest discovered regulator of iron metabolism and is a molecule that safely retains excess intracellular iron in the cores of its shells.^14^ Iron can induce oxidative damage to blood components and the arterial wall, thereby initiating the atherosclerotic process. Medical evidence indicates that elevated body iron stores may serve as a risk factor for the development of atherosclerosis. In a Genome-Wide Association Study (GWAS) of 15,666 patients with diabetes, genetically predicted iron concentrations were associated with the risk of CAD.^15^ A study showed inconsistent correlations between different iron metabolism markers and CAD. Increased total iron binding capacity and serum iron were found to have a protective role against CAD in women. However, a high ferritin level was significantly associated with CAD incidence in both men and women.^16^^,^^17^ This result aligns with the previous study. Regarding ferritin, hypoxic conditions, such as Sleep Disordered Breathing (SDB), may upregulate its levels.^18^ In recent years, accumulated evidence has shown that ferritin plays an indispensable role in the development of CAD.^19^, ^20^, ^21^, ^22^ On the one hand, Khalili A et al.^23^ found that serum ferritin levels were significantly correlated with both Oxidation of Low-Density Lipoprotein (OX-LDL) concentrations and CAD scores in the study group. Furthermore, measuring OX- LDL and ferritin could greatly aid in predicting premature CAD. On the other hand, proteomics and molecular biology studies have shown that ferritin levels in arteries are increased in diseased tissues, which further supports the link of ferritin to CAD/MI.^5^ A study demonstrated that ferritin modulates the progression of atherosclerosis by regulating the expression of matrix Metalloproteinases (MMPs) and interleukins. Silencing ferritin impeded the advancement of atherosclerosis.^24^ Ferritin served as an independent risk factor for arterial stiffness in a study population and significantly influences the relationship between inflammation and Pulse Wave Velocity (PWV).^25^ These also align with the authors’ conclusions. In the present study, the authors found that CAD patients had high mortality with increasing ferritin levels. Specifically, the risk of all-cause mortality was 167.4 % higher compared to patients in the high ferritin group.

However, several studies present differing opinions. Auer J et al.^26^ suggested that in patients undergoing coronary angiography, higher ferritin concentrations and transferrin saturation levels were not correlated with an increased extent of coronary atherosclerosis. Another study indicated that their findings do not support the role of biochemical or genetic markers of iron stores as predictors of CAD risk or its thrombotic complications. They did not find a statistical difference between ferritin and CAD.^27^ High ferritin levels before and after menopause were also not associated with CAD.^28^ Grammer TB et al.^29^ found that serum iron, transferrin saturation, soluble transferrin receptor, and ferritin had J-shaped associations with cardiovascular and total mortality in stable CAD patients undergoing angiography. These discrepancies could be attributed to the varied geographic locations of the subjects and the smaller sample size. Additionally, their follow-up period was shorter than that of the present study.

Also, Grammer TB et al.^30^ showed that both low hemoglobin and iron depletion were independently associated with angiographic CAD. A further study in geriatric patients revealed that serum ferritin, a marker of intracellular iron, significantly correlated with CAD, though this correlation was not independent. Given the prevalence of iron deficiency in elderly patients, the iron hypothesis should be expanded to encompass both iron deficiency and iron toxicity.^17^ This warrants further investigation and is a critical area for future research.

## Study limitations

In the present study, there were several limitations. First, the authors only collected the baseline level of ferritin and did not analyze dynamic changes over the study period. Second, the enrolled patients were all from a single cohort, and ferritin is not a routine test, potentially causing selection bias. Lastly, larger population studies are needed to create more homogeneous groups with balanced patient numbers and comparable characteristics.

## Conclusion

In conclusion, this study indicates that baseline ferritin is an independent predictor of all-cause death in CAD patients. Internists should place greater emphasis on controlling their ferritin levels to reduce the risk of all-cause mortality and improve the prognosis.

## Ethics approval and consent to participate

This study was performed in line with the principles of the STROBE Statement. The PRACTICE study (Identifier: NCT05174143) is a large, single-centre, prospective cohort study. The ethics committee of the First Affiliated Hospital of Xinjiang Medical University approved this research. All patients provided informed consent to participate.

## Authors’ contributions

Zhi-Hui Jiang made substantial contributions to the study conception and design and to the drafting and critical revision of the manuscript for important intellectual content. Ming-Ming Lyu, Yu-Juan Feng, Ting-Ting Wu, Ying Pan, Chang-Jiang Deng, Yi Yang, and Xiao-Xia Guo made substantial contributions to the study by data and sample collation and checking. Xiang Xie made substantial contributions to the study conception and design and to the drafting and critical revision of the manuscript for important intellectual content, including study supervision. All authors agreed to the publication of this work.

## Funding

This work was supported by the National Natural Science Foundation of China (82,260,070).

## Declaration of competing interest

The authors declare no conflicts of interest.
